# Immunization of Foals with Equine Rotavirus A Vaccine Can Stabilize or Increase Rotavirus-Specific Neutralizing Antibody Titers

**DOI:** 10.3390/v18050556

**Published:** 2026-05-13

**Authors:** Lianne G. Eertink, Olivia Jacob, Emma N. Adam, Allen E. Page, Dan Wang, Feng Li

**Affiliations:** Maxwell H. Gluck Equine Research Center, Department of Veterinary Science, University of Kentucky, Lexington, KY 40503, USA

**Keywords:** equine rotavirus A, vaccination, virus-neutralizing antibody titers, foals

## Abstract

Despite a monovalent G3P[12] (‘G3’) vaccine being available for horses, equine rotavirus A (ERVA) is still the predominant infectious pathogen causing diarrhea in foals in the United States of America (U.S.). Previous research has shown that maternal neutralizing antibody (NAb) titers are too high and will interfere with the vaccination of foals at 30 and 45 days of age. We aimed to determine if it is possible to increase NAb titers in foals through vaccination before they are vulnerable to ERVA infection. We immunized two foals with the commercially available vaccine (G3) at solely three months of age and seven foals at both two and three months of age, respectively. Two mock foals were vaccinated with saline buffer in this study. The dams of these foals were not vaccinated during their gestation period. All pre-vaccination G3 and G14P[12] (‘G14’) NAb titers in this foal cohort were 256 or lower. Following vaccination, NAb titers in foals were increased up to 1024 against G3 and up to 512 against G14 viruses, respectively. Interestingly, ERVA NAb titers either increased or stabilized in immunized foals depending on the pre-vaccination NAb titer, which contrasts with unvaccinated foals showing a rapid decline in NAb titers over time.

## 1. Introduction

Equine rotavirus A (ERVA) is the leading infectious pathogen causing diarrhea in foals within the United States of America (U.S.) [1]. The severity of disease in foals depends often on the foal’s age, with younger foals being more severely affected than foals that are older in age. The predominant clinical sign that can be observed is watery, but not bloody, diarrhea; however, other clinical signs such as signs of colic, abdominal distention, appetence and a dull appearance can be noticed as well, which has a significant impact on the animal’s welfare [2]. With appropriate treatment, mortality of foals with ERVA infections is low. However, due to the extensive cost of this treatment and the highly infectious nature of ERVA which often leads to multiple foals on the same farm needing this treatment, ERVA is still a significant financial burden to the equine industry. Fortunately, since 1996, a monovalent killed G3P[12] (‘G3’) vaccine has been available in the U.S., which is used to vaccinate pregnant mares at 8, 9, and 10 months of gestation. These maternal antibodies are then passively transferred to the foals through the colostrum. In our previous study, we demonstrated that these antibodies steadily decline after birth, with, in general, the lowest amount of ERVA-neutralizing antibodies (NAbs) measured at four months of age, with some fluctuations between individual foals [3]. Although often less severe than infection at younger ages, ERVA infection in foals from vaccinated dams is most commonly observed after two months of age [4]. One way to counteract this would be by providing the foals with active immunity through vaccination before the ERVA maternal antibodies are at a critical low level. However, vaccination of young animals is often controversial due to maternal antibody interference. In general, vaccinating foals before six months of age has shown itself to be ineffective. Vaccinating foals using an influenza vaccine with two different time schedules (12, 16 and 32 weeks or 24, 28 and 44 weeks of age) did show that titers could be increased with both schedules; however, the later time schedule showed to be more effective by inducing a stronger and more durable immune response [5]. For foals to develop active immunity, their own immune system needs to be activated by rotavirus antigen, so memory T and B cells are generated. However, maternal antibodies in foals prevent exposure of the foal’s naïve immune system to the vaccine antigens, most likely through vaccine clearance [6]. A previous study by Dwyer [7] showed that ERVA vaccination of foals born to vaccinated dams at 30 and 45 days of age did not result in a difference in ERVA NAb titers between the vaccinated group of foals and their age-matched unvaccinated controls. Both groups decreased steadily in titers over time, which confirmed that at this age maternal antibodies are still too high and will interfere with vaccination. Alternatively, it has been proposed that it is not solely a maternal antibody interference, but also the immaturity of the foal’s immune system that prevents foals from responding to vaccination before six months of age. At two to three months of age, foals are fully capable of producing IgM (immunoglobulin M), IgG1, IgG3, IgG5, and IgA. However, the production of IgG4, IgG7, and IgE is lower in comparison to the production in adult horses. Additionally, there is evidence that T helper (Th) cell functionality and cytokine production, which are important for proper B cell stimulation, are impaired for at least three months after birth [8]. Therefore, this study aimed to determine whether immunization at a later timepoint following birth (two and/or three months of age) can avoid maternal antibody interference for viral antigen presentation, which in turn can stimulate the development of higher titers of ERVA NAbs.

## 2. Materials and Methods

### 2.1. Animals and Sample Collection

A total of 22 horses, of which 11 were mares and 11 were their foals, were involved in this study (Table 1). These horses were housed at the University of Kentucky’s research farm for the duration of this study. Four of the mares were home bred, meaning that they were born at the research farm, while the other mares were donated to the research farm. No ERVA vaccination or natural exposure history is available for the donated mares prior to their arrival to the research farm. Four years prior to the experiment, the research farm experienced rotavirus A infection among their foals. Some mares were vaccinated the previous year for ERVA using the commercially available vaccine. All mares were on the research farm for four years or longer at the start of the experiment. The mares in this study were not vaccinated during the gestation of the foals in this study. However, it is possible that these mares experienced previous natural exposure or ERVA vaccination; therefore, these mares might not have been completely naïve to ERVA and could have passed ERVA maternal antibodies to their foals after birth.

**Figure 1:**
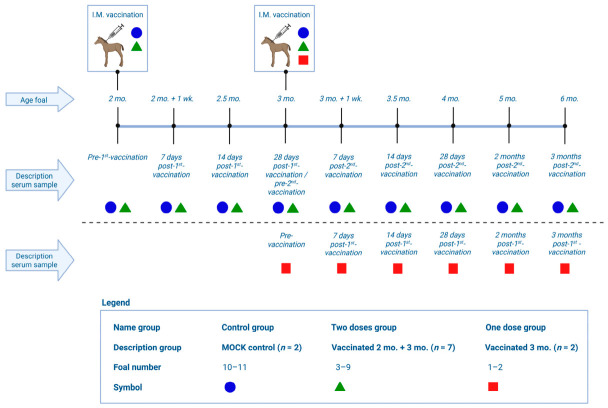
Experimental design: A total of 11 foals were vaccinated using a saline buffer (MOCK; *n* = 2), the ERVA vaccine at 2 and 3 months of age (*n* = 7), or solely at 3 months of age (*n* = 2). Serum samples were taken at the timepoints described in the figure. I.M. = intramuscular, mo. = months, wk. = week. Created with BioRender.com.

Foals were divided into three groups: foals mock-vaccinated intramuscularly using saline buffer injection (control group; *n* = 2), foals vaccinated intramuscularly off-label using the Zoetis (Parsippany, NJ, USA) monovalent G3 vaccine at both two and three months of age (two-doses group; *n* = 7), and foals vaccinated intramuscularly off-label using the Zoetis monovalent G3 vaccine at solely three months of age (one-dose group; *n* = 2). For four of the eleven mares, serum was collected pre-foaling, while for the other seven mares, post-foaling serum was collected to establish approximate ERVA NAbs in these mares. Vaccination of foals and subsequent serum collection were carried out according to the schedule described in Figure 1. Briefly, pre-vaccination serum was collected from foals, followed by 7, 14, and 28 post-vaccination timepoints. In addition, serum from foals was collected at two and three months after the last vaccination.

### 2.2. Sample Processing and Preparation

Blood samples were collected by jugular venipuncture with a Vacutainer collection system without coagulant and an 18-gauge needle. Shortly after, collection samples were transported to the laboratory, followed by a brief centrifugation. The separated serum fractions were aliquoted and stored at −20 °C until further use.

### 2.3. Virus Neutralization Assay (VNA)

All the serum samples described in Figure 1, together with the serum samples collected from the mares (*n* = 104), were analyzed by a cell-based VNA against both G3 and G14P[12] (‘G14’). MA104 NxV cells, which are an engineered cell line with a reduced IFN response for increased rotavirus growth, were plated onto 96-well plates and incubated for three days at 37 °C under 5% CO_2_ conditions [9]. The same incubation conditions were maintained throughout the experiment. After three days, the plates were confluent and either 300 FFU/50 μL G3 or 300 FFU/50 μL G14 virus was activated using 10 μg/mL TPCK-trypsin for one hour. Serum samples were two-fold serially diluted starting at a dilution of 1:4 up to 1:4096. A control only containing virus without serum was included on each plate to assure the proper virus dose used in the VNA. After activation, 50 μL of the virus was mixed with 50 μL of the previously made serum dilutions and incubated for one hour. Hereafter, this mixture was added to the washed cells and incubated for another hour after which the cells were washed again, and virus growth media was added. Plates were incubated for 18 h after which they were fixed using 80% acetone. After washing, plates were immunostained, first for one hour with the commercially available primary antibody (Rotavirus Polyclonal Antibody; Invitrogen, Carlsbad, CA, USA), followed by an additional one-hour incubation with the commercially available secondary antibody (Rabbit anti-Goat IgG (H + L) Secondary Antibody, FITC; Invitrogen, Carlsbad, CA, USA). After the final wash, plates were read using a fluorescent microscope to determine NAb titers, which are expressed as the reciprocal of the highest dilution that resulted in a 50% reduction in FFUs. All the serum samples were assayed three times in independent experiments.

### 2.4. Data Analysis

All graphs were made using GraphPad Prism 10 (GraphPad Software, LLC, Boston, MA, USA), statistical analyses were done using SAS 8.3 software (SAS Institute Inc., Cary, NC, USA). All statistical values and graphing of the VNA data were done using Y = log2(Y) transformed data due to the heteroscedasticity. The differences between VNA titers of different groups at the different timepoints were explored using Analysis of Variance (ANOVA) by a PROC MIXED model with the research groups, the foal’s age and the interactions between these two variables (group x foals’ age) as fixed effects. *p*-values were calculated based on the differences in the least square means (LSM) and are considered significant at *p* ≤ 0.05.

## 3. Results

### 3.1. Safety

Eight of the nine foals that were vaccinated using the Zoetis vaccine did not show any clinical signs or adverse reactions in the days after vaccination. However, foal 6 did develop a fever and behaved abnormally after the first vaccination. This foal received a dose of flunixin meglumine, a non-steroidal anti-inflammatory drug, which could have affected his response to the vaccine. The pre-vaccination peripheral blood mononuclear cell (PBMC) count of this foal was markedly lower (6.01 × 10^6^ cells/mL) in comparison to the other foals within the experiment at this timepoint. Foal 6 did not experience any adverse reactions during the second vaccination a month after the first vaccination.

### 3.2. Mare ERVA NAb Titers and Foal Pre-Vaccination ERVA NAb Titers

**Figure 2:**
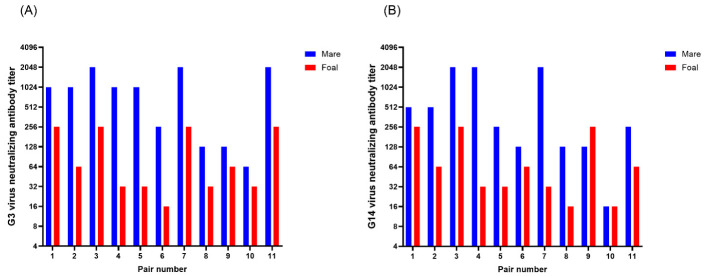
Mare pre- or post-foaling G3 virus neutralizing antibody (NAb) titer and foal pre-vaccination G3 NAb titer (**A**), and mare pre- or post-foaling G14 virus NAb titer and foal pre-vaccination G14 NAb titer (**B**). Pair number is the mare–foal pair number and corresponds to the mare’s and foal’s number.

Despite these mares not being vaccinated during the gestation of the foals in this experiment, most of the mares still had ERVA NAbs around the time of foaling (Figure 2). Mares’ G3 NAb titers ranged between 64 and 2048, while G14 NAb titers ranged between 16 and 2048. Most of the foals had considerable G3 and G14 NAb titers before their first vaccination (pre-vaccination serum sample) which was taken at two months of age for foals 3–11 and at three months of age for foals one and two. Both the G3 NAb titers and G14 NAb titers of pre-vaccination serum samples of foals ranged between 16 and 256. For both G3 and G14 NAb titers there is at least one or more foals with a pre-vaccination titer of 16, 32, 64, and 256, but there were no foals that had a pre-vaccination titer of 128. Therefore, all foals had some level of ERVA maternal antibodies pre-vaccination.

### 3.3. Foal ERVA NAb Titers Following Vaccination

**Figure 3:**
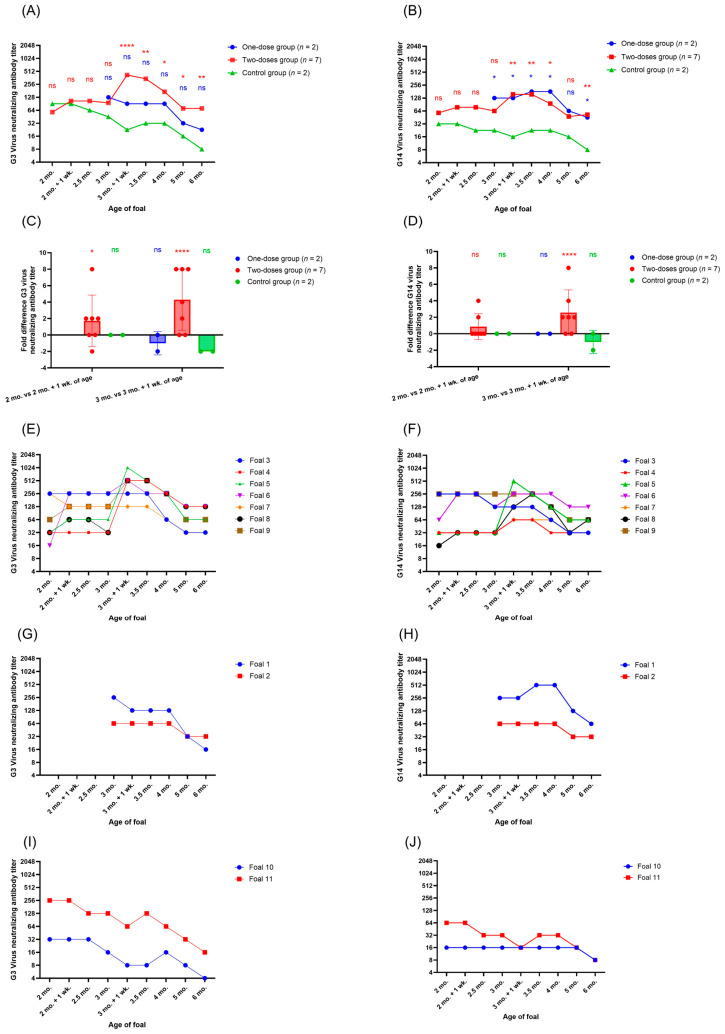
Mean G3 (**A**) and G14 (**B**) neutralizing antibody (NAb) titers in serum samples per foal research group based on log2-transformed data (Y = log2(Y)). The statistical differences indicated are between the one-dose group and control group (blue), and the two-dose group and control group (red) at each timepoint. NAb titer difference for G3 (**C**) and G14 (**D**) between the pre-vaccination serum sample at 2 months of age and at 7 days post the first vaccination for the two-dose and control research groups (2 months of age). Furthermore, the difference in NAb titers between the pre-vaccination serum samples at 3 months of age and 7 days post vaccination, which includes the second (mock) vaccination for the two-dose and control research groups, and the first vaccination for the one-dose research group, is shown. Statistics indicate a difference in NAb titers within a group between pre- and post-(mock)vaccination. G3 and G14 NAb titers for individual foals within the two-dose research group (respectively (**E**) and (**F**)), the one-dose research group (respectively (**G**) and (**H**)), and the control group (respectively (**I**) and (**J**)). Statistical differences in graphs A and B are between the research group and the control group. * *p*-value ≤ 0.05, ** *p*-value ≤ 0.01, **** *p*-value ≤ 0.0001, ns is not significant (*p* > 0.05).

The ERVA NAb titers of the control group decreased, as expected, steadily over time up to three months and one week of age, after which a minor increase in titers was observed for both G3 and G14, followed by a further decline in ERVA NAb titers (Figure 3A,B). These figures include the data of all foals; no foals or datapoints were excluded in the analysis. On average, a minor increase in ERVA NAb titers (1.7-fold increase for G3, 0.9-fold increase for G14) was observed after the first vaccination of the foals within the two-dose research group (Figure 3C,D). However, a much more pronounced difference was observed between the pre-second-vaccination serum samples and the seven-day post-second-vaccination samples of the foals within the two-dose group: a 4.3-fold increase for G3 NAb titers and a 2.6-fold increase for G14 NAb titers. For the two foals that were vaccinated only once at three months of age (one-dose group), a negligible decrease in one-fold was observed between their pre-vaccination sample and their seven-day post-vaccination G3 NAb titer, and no difference in G14 NAb titers was observed. It is important to note that the ERVA NAb titers of the one-dose group did not decrease with time as seen in the control group. Foals started out with NAb titers that, on average, did not differ more than 2- to 4-fold between groups at pre-vaccination. The difference between G3 NAb titers at seven days post-second-vaccination of the two-dose group and the control group was, on average, 8.4-fold and 6.6-fold for G14 NAb titers, respectively. The difference between the one-dose group and the control group at seven days post-vaccination was 4.0-fold for G3 NAb titers and 6.0-fold for G14 NAb titers. At six months of age, the vaccinated foals of both groups still had higher G3 and G14 NAb titers than the control group. Compared to the control group, the two-dose group’s G3 and G14 NAb titers were 3.1-fold and 2.7-fold higher, respectively. Compared to the control group, the one-dose group’s G3 and G14 NAb titers were 1.5-fold and 2.5-fold higher, respectively.

When looking at the individual foals within each group, some clear differences can be observed between foals. Within the two-dose group, both foals 3 and 7 started out with a G3 NAb titer of 256, and neither increased in titer throughout the experiment (Figure 3E,F). However, their G3 NAb titers also did not decrease steadily as seen in the control foals, but rather they stabilized at a titer between 128 and 256 up to 3.5 months of age. Similarly, foals 3 and 9 started out with a G14 NAb titer of 256 and neither foal increased in titer throughout the experiment, but both foals had a stable titer between 128 and 256 up to 3.5 months of age for foal 3, and 4 months of age for foal 9. Interestingly, the foal with the lowest G3 NAb titer (16) at the start of the experiment, foal 6, is the only foal that responded tremendously to the first vaccination by increasing 8-fold in titer seven days after the first vaccination. This is also the foal that developed a fever and abnormal behavior after the first vaccination but behaved normally throughout the rest of the experiment. The foals that increased the most in G3 NAb titer at seven days after the second vaccination (foals 4, 5, 8, and 9) all had G3 NAb titers between 32 and 64 at the start of the experiment. Foals 5 and 8 increased the most in G14 NAb titers seven days after the second vaccination and started out with G14 NAb titers of, respectively, 32 and 16 at the start of the experiment. However, foals 4 and 7 barely increased in G14 NAb titers throughout the experiment despite having G14 NAb titers of 32 at the start of the experiment. Both foals 1 and 2 within the one-dose group had relatively stable G3 and G14 NAb titers up to four months of age (Figure 3G,H). Foals 10 and 11 were both in the control group; their G3 NAb titers decreased up to three months and one week of age, while between then and four months of age, a negligible increase in G3 titers can be observed, after which they decreased further (Figure 3I). The G14 NAb titer of the control foal 10 was very low throughout the experiment and did not decrease further until five months of age (Figure 3J). The G14 NAb titer of foal 11 was slightly higher at the start of the experiment than that of foal 10 and decreased over time.

## 4. Discussion

Overall, this study showed that it is possible to generate an immune response in foals younger than six months of age in response to ERVA vaccination. However, the foal’s pre-vaccination ERVA NAb titers might be of great influence on the extent of the effective immune response. The major limitation of this study is the limited number of foals, especially in the one-dose and control groups. Furthermore, four years prior to the experiment, the research farm experienced an outbreak of ERVA among their foals; however, it is expected that no ERVA was present on the farm during the current study, to the best of our knowledge. No foals within our study or any other herds on the research farm developed diarrhea related to ERVA infection during the year of the study. Therefore, we strongly expect that the foals within this study were not naturally exposed to ERVA for the duration of the study.

The foal with the lowest G3 NAb titer (16) at the start of the study (foal 6) responded tremendously to the first vaccination by increasing its G3 NAb titer by 8-fold. However, this foal also developed a fever and behaved abnormally after vaccination, for which he was treated with flunixin meglumine. This medication could have dampened the immune response resulting from the vaccination. It is not clear if this response was due to the vaccination or due to an underlying, unrelated medical condition. Even though this vaccine has proven to be safe for use in adult horses (*n* = 627) since no adverse reactions were observed [10], the safety of this vaccine for use in foals needs to be further investigated. In the study of Dwyer [7], one of the fifty foals that were vaccinated at 30 and 45 days of age developed a fever of 39.5 °C after vaccination, but no other adverse effects were observed.

As the foal’s immune system is less mature than that of adult horses, foals might respond differently to vaccination. Up to three months of age, Th2 activity as measured by IL-4 concentrations is barely detectable. This indicates that Th2 cells might not be mature at this age, which is a possible reason for reduced immune responses to vaccination in foals, since Th2 cells are essential in stimulating B cells [8]. A larger number of foals (>100) needs to be studied to confirm the safety of using this vaccine in foals, as only larger numbers can identify rare adverse effects with a prevalence of less than 1%.

We were not able to collect pre-foaling samples from all the mares; therefore, for several mares, the post-foaling samples were collected. However, this should not have affected the ERVA NAb titers measured around the time of foaling, as mare NAb titers remain very stable for a prolonged period of time [3]. Even though the mares were not vaccinated during the gestation of the foals in this experiment, they were not naïve to ERVA. This was expected since all mares in this study were present on the farm during the ERVA outbreak, which was about four years prior to this experiment. Additionally, some of the mares might have been vaccinated in previous years by the research farm’s veterinarians or prior to their donation to the University of Kentucky.

Overall, we showed that vaccination of foals at two and/or three months of age can stabilize or increase their ERVA NAb titers. A sensitivity analysis excluding foal 6 from the group data was not performed, which is a limitation of this study. Foal 6 was treated with flunixin meglumine after the first vaccination, which could have altered the foal’s immune response to the vaccine. However, since foal 6 had a big increase in ERVA NAb titer after the first dose but the overall difference between the two-dose group and the control group was not significant at that timepoint, the inclusion of foal 6 did not change the significance at that timepoint. Similarly, since foal 6 did not increase in titer at the second dose, it could be expected that the inclusion of the data of this foal could have reduced the significance of the two-dose group as a whole after the second dose. However, since the difference between the two-dose group and the control group is very significant at this timepoint, we do not expect that it had a major effect on the group data. A big difference in how well foals responded based on their pre-vaccination NAb titers was observed. Based on this exploratory study, foals with a pre-vaccination NAb titers of 64 or higher seemed to increase in NAb titer only slightly or stabilized their NAb titer throughout the study up to 3.5–4 months of age. It is unclear how foals with a pre-vaccination titer of 128 or 512 and higher would respond to vaccination since there were no foals in this study with those NAb titers. We expect that foals with a pre-vaccination NAb titer of 128 would respond similarly to foals with a pre-vaccination NAb titer of 64 or 256. In our study, there are no foals with a pre-vaccination NAb titer higher than 256, so we cannot discuss how foals with such titers might respond to ERVA vaccination. We expect that at some level of NAb titer, the antibody interference would be too high for the foals to be able to respond to the vaccination. Further research needs to include foals with higher ERVA titers to determine the cutoff value at which vaccination of foals is not effective. The foals that responded best to vaccination, in regard to the highest increase in NAb titers post-vaccination, were the foals that had a pre-vaccination NAb titer of 32 or lower. This is in line with the study of Van Maanen, Bruin [5], who showed a close relationship between influenza maternal antibody titers and the immune response of foals after influenza vaccination. Foals with lower titers responded better to vaccination than foals with higher pre-vaccination titers. We have shown that ERVA vaccination of foals can lead to an increase in NAb titers. Further research is needed to establish the function of this vaccine in foals born to vaccinated dams. If the foal’s immune responses are similar to those of the foals in this study, then this vaccine could be useful in situations where dams are vaccinated, and outbreaks occur in older foals.

However, it needs to be determined how well foals from vaccinated dams can be vaccinated at these ages. Based on 18 foals from vaccinated dams within our previous study, we showed that the majority of the foals had a G3 NAb titer of 256 or lower at two months of age. G3 NAb titers of these foals ranged between 128 and 2048, with 13 out of 18 foals having a G3 NAb titer of 256 or lower. For G14, a range of 16 to 512 NAb titers was observed, with 15 out of 18 foals having a G14 titer of 256 or lower at two months of age [3]. Therefore, it might be feasible to vaccinate these foals to at least stabilize their NAb titers; however, it is unlikely that these foals will increase in NAb titer after vaccination at this age. For the best results, it might be recommended to test NAb titers before vaccination to ensure a low enough NAb titer. However, since there is no quick field test available to test this, it might not be practical for breeders.

In this study we showed that G3 and G14 NAb titers were maintained or increased between 64 and 1024 and 64 and 512, respectively, after vaccination. There are no studies done in foals or any other animals in which the protective ERVA NAb titers are determined. However, based on a human study, it has been shown that a NAb titer of 128 or higher is protective [11]. There might be some fluctuations between humans and foals, but it is expected that the protective NAb titer in foals is within proximity of 128. Therefore, we expect that increasing or maintaining foals’ ERVA NAb titers to 128 or higher after vaccination should inhibit infection or at least reduce disease severity. Furthermore, the immunity generated in foals is active immunity in contrast to the immunity passed by the mares, which is passive; therefore, foals might be better equipped to respond to future ERVA challenges after vaccination. Non-neutralizing antibodies were not measured in this study but might offer some additional protection. Non-neutralizing antibodies against the pores of the inner capsid protein, VP6, have been shown to provide protection by intracellularly blocking the release of replicated mRNA through capsid pores [12,13].

The increase in ERVA NAb titers after vaccination of foals decreased relatively quickly within two months of time. This is in line with other studies in which foals are vaccinated before six months of age. Vaccination of foals for influenza at six and ten weeks of age and for a third time between three and five months of age did not result in long-lasting antibodies in foals. At four months after the last vaccination, no influenza antibodies were detected in 30 out of 31 foals [14]. In another study, foals were vaccinated at 90, 120, and 180 days of age for Eastern equine encephalomyelitis, Western equine encephalomyelitis, West Nile virus, equine influenza, tetanus toxoid, and equine herpesvirus (EHV) 1 and 4. For some of these pathogens, but not all, a transient increase in specific antibody titers between 90 and 120 days of age was observed. This indicates that vaccination of foals can be effective for certain pathogens, although the response is in general transient [15]. Therefore, ERVA vaccination of foals might not prevent infection in the long-term. However, delaying the decrease in ERVA NAbs over time might benefit foals since the severity of clinical signs decreases as foals age. Thus, vaccination of foals might be able to provide additional protection during a time at which foals can still become severely affected by ERVA infection. Further research is necessary to determine the effect of a second or third vaccination at 4–6 months of age and its effect on the longevity of ERVA NAbs in foals.

## 5. Conclusions

In conclusion, we have shown that ERVA vaccination using either two doses at two and three months of age or one dose at three months of age can increase or maintain ERVA NAb titers over time up to 3.5–4 months of age. Based on the individual foal’s ERVA NAb immune responses, it is likely that the pre-vaccination NAb titers are of great influence on the extent of the foal’s immune response to ERVA vaccination. In this exploratory study, foals with a pre-vaccination G3 NAb titer between 64 and 256 increased only slightly in NAb titer or remained stable in NAb titers in comparison to the control foals. However, foals with a pre-vaccination G3 NAb titer of 32 or lower responded with a substantial increase in NAb titers at either the first or the second vaccination. Further studies are needed to validate the exact pre-vaccination ERVA NAb titers needed for foals to be able to optimally respond to vaccination. Although controversial, it seems that it is possible to vaccinate foals before six months of age for ERVA. This could be relevant during outbreaks in foals from dams that have not been vaccinated, or in foals from vaccinated dams with low enough maternal ERVA NAb titers at two and or three months of age.

## Figures and Tables

**Table 1:** Overview of foals and mares involved in the experiment.

Foal: # ^1^	Foal: Sex	Foal: Breed	Mare: #	Mare: Breed	Mare: Age at the Time of the Experiment	Mare: Donated/Homebred ^2^	Mare: Years on the Research Farm at the Time of the Experiment
1	Female	Mixed	1	Mixed	9	Homebred	9
2	Female	Mixed	2	Mixed	9	Homebred	9
3	Male	Mixed	3	Thoroughbred	18	Homebred	18
4	Male	Mixed	4	Thoroughbred	13	Donated	5
5	Female	Mixed	5	Thoroughbred	19	Donated	6
6	Male	Mixed	6	Thoroughbred	9	Donated	4
7	Male	Mixed	7	Thoroughbred	13	Donated	4
8	Male	Mixed	8	Mixed	6	Homebred	6
9	Female	Mixed	9	Thoroughbred	14	Donated	5
10	Female	Mixed	10	Thoroughbred	7	Donated	4
11	Male	Mixed	11	Thoroughbred	18	Donated	6

^1^ # = number; ^2^ Note “homebred” means that the mare was born on the university’s research farm.

## Data Availability

The original data presented in this work are included in this article. Further inquiries and communications on the data and other materials used in this study can be sent to the corresponding author of this article.

## Abbreviations

The following abbreviations are used in this manuscript:

ERVA	Equine rotavirus A
U.S.	United States of America
NAbs	Neutralizing antibodies
G3	G3P[12]
G14	G14P[12]
Ig	Immunoglobin
Th cell	T helper cell
VNA	Virus neutralization assay
ANOVA	Analysis of Variance
LSM	Least square means
IACUC	Institutional Animal Care and Use Committee
